# COVID-19 and cap: What changed in training and practice for early career child/adolescent psychiatrists?

**DOI:** 10.1192/j.eurpsy.2021.140

**Published:** 2021-08-13

**Authors:** A. Seker

**Affiliations:** Child And Adolescent Psychiatry, Erciyes University Hospital, Kayseri, Turkey

**Keywords:** child and adolescent psychiatry training, child psychiatry pandemic, early career child and adolescent psychiatry, child psychiatry and COVID-19

## Abstract

The Covid-19 pandemic has transformed the world since the beginning of 2020 and many aspects of health care provision has changed dramatically. Despite not being regarded as a frontline field at first look, Child and Adolescent Psychiatry (CAP) has been highly impacted by the pandemic as a fundamentally biopsychosocial branch. CAP specialty training has also become a different experience due to the reprioritization of health care services along with the restricting rules of the ‘new normal’. In this symposium presentation, we will discuss the effects of the Covid-19 pandemic on the training and practice of CAP specialty trainees and early career specialists through the results of the ‘Early Career Psychiatrists in Europe during COVID-19 outbreak’ survey study, organized by the EPA and EFPT. The survey covers different areas including reorganization of training/practice during the pandemic, personal experiences with Covid-19 and adoption of Telepsychiatry practices. This pan-European study is expected to shed a light on the emerging issues for young doctors in CAP to plan necessary improvements on a European scale.

**Disclosure:**

No significant relationships.

